# Longitudinal relationship between intramuscular adipose tissue of the quadriceps and activities of daily living in older inpatients

**DOI:** 10.1002/jcsm.12842

**Published:** 2021-10-26

**Authors:** Naoki Akazawa, Masaki Kishi, Toshikazu Hino, Ryota Tsuji, Kimiyuki Tamura, Akemi Hioka, Hideki Moriyama

**Affiliations:** ^1^ Department of Physical Therapy, Faculty of Health and Welfare Tokushima Bunri University Tokushima Tokushima Japan; ^2^ Department of Rehabilitation Kasei Tamura Hospital Wakayama Japan; ^3^ Life and Medical Sciences Area, Health Sciences Discipline Kobe University Kobe Japan

**Keywords:** Intramuscular adipose tissue, Activities of daily living, Older inpatients, Muscle mass, Quadriceps, Longitudinal study

## Abstract

**Background:**

The longitudinal relationship between intramuscular adipose tissue of the quadriceps and activities of daily living (ADL) in older inpatients remains unclear. This study aimed to examine whether decrease of intramuscular adipose tissue of the quadriceps in older inpatients is related to the recovery of ADL than increase of muscle mass.

**Methods:**

This longitudinal study included 202 inpatients aged ≥65 years [median age: 83.0 (77.0–88.0), 56.4% female]. Recovery of ADL during hospital stay was assessed using the change in Barthel index (BI) score (i.e. BI score at discharge minus BI score at admission) and BI score at discharge. Intramuscular adipose tissue and muscle mass of the quadriceps were assessed using echo intensity and muscle thickness on ultrasound images, respectively. Higher echo intensity indicates greater intramuscular adipose tissue. Multiple regression analysis was performed to identify the factors independently associated with the change in BI score and BI score at discharge. Changes in quadriceps echo intensity and thickness and subcutaneous fat thickness of the thigh, quadriceps echo intensity and thickness, and subcutaneous fat thickness of the thigh at admission, age, sex, days from onset disease, BI score at admission, and disease were set as independent variables.

**Results:**

The means of the change in quadriceps echo intensity and thickness were −2.3 ± 15.7 and 0.1 ± 0.4 cm, respectively. The median of the change in BI score was 15.0 (0.0–30.0). The quadriceps echo intensity at discharge was significantly lower than at admission (*P* = 0.043). The quadriceps thickness (*P* = 0.004) and BI score at discharge (*P* < 0.001) were significantly higher than those at admission. Change in quadriceps echo intensity was independently and significantly associated with the change in BI score (β = −0.25, *P* = 0.006) and BI score at discharge (β = −0.18, *P* = 0.006). In contrast, change in quadriceps thickness was not independently and significantly associated with the change in BI score (β = 0.09, *P* = 0.244) and BI score at discharge (β = 0.06, *P* = 0.244).

**Conclusions:**

Our study indicates that a decrease of intramuscular adipose tissue of the quadriceps is related to the recovery of ADL than an increase of muscle mass in older inpatients. Intramuscular adipose tissue of the quadriceps in older inpatients is considered to be a predictor for the recovery of ADL, and intervening for intramuscular adipose tissue may be important for improving ADL in older inpatients.

## Introduction

Recent studies^1^, ^2^, ^3^, ^4^ reported that intramuscular adipose tissue increases with aging. In addition, an increased intramuscular adipose tissue has been reported to be related to a decreased muscle strength^5^, ^6^, ^7^; decreased sit‐up–sit‐down,^5^, ^8^ stair ascent–stair descent,^9^ and gait abilities^6^, ^10^, ^11^; onset hip fracture^12^; and mortality^13^, ^14^ than the loss of muscle mass. Therefore, an increase of intramuscular adipose tissue is recognized as a severe problem in old age.

More recently, we reported that a greater intramuscular adipose tissue of the quadriceps in older inpatients at post‐acute hospital admission is associated with a worse recovery of activities of daily living (ADL) than the loss of muscle mass.^15^ Based on these findings, a decrease of intramuscular adipose tissue in older inpatients is considered to be related to the recovery of ADL than an increase of muscle mass. However, the longitudinal relationship between intramuscular adipose tissue of the quadriceps and ADL in older inpatients remains unclear. Understanding this longitudinal relationship is to be important in developing an effective approach for improving ADL in older inpatients. This study aimed to examine whether decrease of intramuscular adipose tissue of the quadriceps in older inpatients is related to the recovery of ADL than increase of muscle mass.

## Materials and methods

### Study design and participants

This longitudinal study included older inpatients who were referred to the Department of Rehabilitation at Kasei Tamura Hospital. This hospital has subacute and convalescent rehabilitation wards. Patients aged <65 years or those who lacked data were excluded from the study. A total of 242 inpatients were recruited. Of these, 40 patients aged <65 years (*n* = 34) or who lacked necessary data (*n* = 6) were excluded. Ultimately, 202 older inpatients participated in this study. Rehabilitation therapy, including physical therapy, occupational therapy, and speech and swallowing therapy, was administered to all participants during hospitalization. All participants or their guardians provided informed consent prior to the study, and the study was approved by the ethics committee of our institution.

### Outcome measures

The primary outcome was recovery of ADL during the hospital stay and changes in intramuscular adipose tissue and muscle mass of the quadriceps. We also measured the characteristics of the participants at admission including, disease, age, sex, body weight, height, body mass index, subcutaneous fat mass of the thigh, ADL, swallowing ability, nutritional status, inflammation, comorbidities, number of medications, and number of units of rehabilitation therapy (one unit of rehabilitation therapy = 20 min). ADL, intramuscular adipose tissue and muscle mass of the quadriceps, and subcutaneous fat mass of the thigh were measured not only at admission but also at discharge. The length of hospital stay (days) and days from onset disease were measured at discharge. The length of hospital stay was assessed based on the hospitalization period at Kasei Tamura Hospital. The majority of older inpatients at our hospital were admitted from another acute‐phase hospital. In these patients, the days from onset disease were assessed as the total length of stay in both hospitals.

### Measurement of recovery of activities of daily living

Activities of daily living were assessed using the Barthel index (BI).^16^ Recovery of ADL during hospital stay was assessed based on the change in BI score and BI score at discharge. The BI is widely used in clinical settings and includes ordinal assessment (0–100 points).^16^ Lower BI scores indicate poor ability to perform ADL. The BI consists of 10 items: (1) feeding, (2) moving from a wheelchair to a bed and from a bed to a wheelchair, (3) grooming, (4) toilet action, (5) bathing, (6) walking on a level surface, (7) going up and down stairs, (8) dressing, (9) bowel continence, and (10) bladder continence.^16^ The change in BI score was calculated using the following equation: change in BI score = BI score at discharge − BI score at admission. A higher BI score change indicated a greater improvement in the ability to perform ADL.

### Measurements of intramuscular adipose tissue and muscle mass of the quadriceps and subcutaneous fat mass of the thigh

Transverse ultrasound images were obtained using a B‐mode ultrasound system (NanoMaxx; SonoSite Japan, Tokyo, Japan) with a linear‐array probe (L25n/13–6 MHz; Nanomaxx; SonoSite Japan). The intramuscular adipose tissue and muscle mass of the *rectus femoris* and *vastus intermedius* were assessed based on the echo intensity and muscle thickness.^1^, ^2^, ^3^, ^4^, ^5^, ^6^, ^7^, ^8^, ^10^, ^11^, ^15^, ^17^, ^18^, ^19^ The validity of intramuscular adipose tissue and muscle mass measurements using ultrasound has been confirmed in recent studies using magnetic resonance imaging.^17^, ^18^, ^20^ Images of the *rectus femoris* and *vastus intermedius* were obtained at 30% of the distance from the anterior superior iliac spine to the proximal end of the patella.^4^, ^6^, ^11^, ^15^, ^19^ The participants lay in the supine position with their lower limbs relaxed, while a water‐soluble transmission gel was applied to the skin surface of the thigh. The probe was pressed perpendicularly and lightly against the skin to prevent muscle deformation. All ultrasound images were recorded by the same investigator, who had sufficient training in echo intensity and muscle thickness measurements. Echo intensity was measured in one region of interest in the *rectus femoris* and *vastus intermedius*; the region selected included the maximum possible regions, while avoiding the bone and surrounding fascia.^4^, ^6^, ^11^, ^15^, ^19^ To standardize all echo intensity measurements, the gain status was normalized to the initial setting of the ultrasound system. In addition, the image depth was uniformed at 60 mm for all echo intensity and muscle thickness measurements. The *rectus femoris* thickness was determined as the distance between the superficial adipose tissue‐muscle interface and the deep muscle‐muscle interface,^4^, ^6^, ^11^, ^15^, ^19^ and that of the *vastus intermedius* was determined as the distance between the superficial muscle–muscle interface and the bone‐muscle interface.^4^, ^6^, ^11^, ^15^, ^19^ Echo intensity and muscle thickness were measured using ImageJ 1.49 software (National Institutes of Health, Bethesda, MD, USA).^1^, ^2^, ^3^, ^4^, ^5^, ^6^, ^7^, ^8^, ^11^, ^15^, ^17^, ^18^, ^19^ Echo intensity was determined by performing a computer‐assisted 8‐bit grey‐scale analysis, and the mean echo intensity of the regions of interest was expressed as a value from 0 (black) to 255 (white).^1^, ^2^, ^3^, ^4^, ^5^, ^6^, ^7^, ^8^, ^10^, ^11^, ^15^, ^17^, ^18^, ^19^ Higher echo intensity indicates greater intramuscular adipose tissue.^21^

The echo intensity of the quadriceps was calculated as the mean echo intensity of the *rectus femoris* and *vastus intermedius*. The mean echo intensity of the right and left quadriceps was used in the analysis. The sum of the thickness of the rectus femoris and vastus intermedius was used as a measure of quadriceps thickness. The mean thickness of the right and left quadriceps was used in the analysis. The changes in quadriceps echo intensity and thickness were calculated using the following equations: change in quadriceps echo intensity = quadriceps echo intensity at discharge − quadriceps echo intensity at admission, change in quadriceps thickness = quadriceps thickness at discharge − quadriceps thickness at admission. The methods used for measuring the echo intensity and muscle thickness of the rectus femoris and vastus intermedius in our study group have been reported to have high reliability [intraclass correlation coefficients (1.1) = 0.857–0.959].^19^ The subcutaneous fat mass of the thigh was assessed based on the subcutaneous fat thickness. Subcutaneous fat thickness was determined as the distance between the dermis and adipose tissue interface and the muscle–adipose tissue interface.^4^, ^6^, ^11^, ^15^, ^19^ The mean subcutaneous fat thickness of the right and left thigh was used in the analysis. The change in subcutaneous fat thickness of the thigh was calculated using the following equation: change in subcutaneous fat thickness of the thigh = subcutaneous fat thickness of the thigh at discharge − subcutaneous fat thickness of the thigh at admission.

### Measures of other characteristics

Swallowing ability was assessed using the Food Intake Level Scale.^22^ The Food Intake Level Scale is a 10‐point observer‐rated scale, with higher values indicating better swallowing ability. The inflammatory status was assessed by analysing C‐reactive protein concentration. Nutritional status was assessed using the Geriatric Nutritional Risk Index score.^23^ The Geriatric Nutritional Risk Index score was calculated using the following formula: Geriatric Nutritional Risk Index score = [14.89 × serum albumin (g/dL)] + [41.7 × body weight (kg)/ideal body weight].^23^ The ideal body weight was defined based on a body mass index of 22.0 kg/m^2.^ ^24^ If the body weight/ideal body weight was ≥1.0, the value was recorded as 1.^23^ Comorbidities were evaluated using the updated Charlson comorbidity index score.^25^

### Sample size calculation

A recent study^15^ reported that the effect size (f^2^) of the multiple regression analysis for the change in BI score was 0.295 (in this multiple regression analysis, the quadriceps echo intensity at hospital admission and the other seven variables were independently and significantly associated with the change in BI score). We expected to observe similar effect size in the multiple regression analysis of this study. *A priori* sample size calculation with an effect size (f^2^) of 0.295, power of 0.95, alpha error of 0.05, and number of predictors of 10 to 15 indicated that a sample size of at least 93 to 108 participants was required. Sample size calculations were conducted using G* Power version 3.1.9.2 (Heinrich‐Heine‐Universität Düsseldorf, Düsseldorf, Germany).

### Statistical analysis

All statistical analyses were conducted using SPSS version 24 software (IBM SPSS Japan, Tokyo, Japan). Variables were assessed for normality using the Shapiro–Wilk test. Parametric data are reported as mean ± standard deviation, whereas nonparametric data are expressed as median [interquartile range (IQR)]. Quadriceps echo intensity and thickness, subcutaneous fat thickness of the thigh, and BI score between hospital admission and discharge were compared using paired *t* test or Wilcoxon signed‐rank test. Multiple regression analysis (forced entry method) was performed to identify the factors independently associated with the change in BI score and BI score at discharge. Changes in quadriceps echo intensity and thickness, quadriceps echo intensity and thickness at admission, subcutaneous fat thickness of the thigh at admission, change in subcutaneous fat thickness of the thigh, age, sex (male = 1, female = 0), days from onset disease, BI score at admission, and disease (reference: stroke) were set as independent variables. The echo intensity is influenced by subcutaneous fat thickness.^26^ Based on this finding, we included subcutaneous fat thickness of the thigh as an independent variable. To assess multicollinearity, we used the variance inflation factor. A variance inflation factor value of more than 10 was considered as the presence of multicollinearity. A *P* value of <0.05 was considered to indicate statistical significance. In addition, we calculated the effect size (f^2^) of the multiple regression analysis using the following equation: *R* ^2^/ (1 − *R* ^2^).^27^ The statistical power of the analysis based on f^2^, an alpha error of 0.05, total sample size, and number of predictor variables was calculated using G* Power version 3.1.9.2.

## Results

The medians (IQR) of the length of hospital stay and days from onset disease of the participants were 71.0 (48.8–98.3) days and 89.5 (62.8–119.0) days, respectively. Diseases found among the participants were stroke (*n* = 35), fracture (*n* = 82, including hip fracture, compression fracture, pubic fracture, and other fracture), pneumonia (*n* = 31), and others (*n* = 54, including heart disease, kidney disease, chronic obstructive pulmonary disease, diabetes, cancer, dehydration, and urinary tract infection). *Table* 1 shows characteristics of participants at hospital admission.

**Table 1:** Characteristics of participants at post‐acute hospital admission (*n* = 202)

Characteristics	
Age, years	83.0 (77.0–88.0)
Sex, male/female	88 (43.6)/114 (56.4)
Height, cm	152.0 (147.0–161.0)
Body weight, kg	47.9 ± 10.6
Body mass index, kg/m^2^	20.3 ± 3.8
Disease	
Stroke	35 (17.3)
Fracture	82 (40.6)
Pneumonia	31 (15.3)
Others	54 (26.7)
Quadriceps echo intensity (grey‐scale range, 0–255)	83.8 ± 20.5
Quadriceps thickness, cm	1.2 ± 0.5
Subcutaneous fat thickness of the thigh, cm	0.4 (0.3–0.5)
Food Intake Level Scale	8.0 (7.0–8.0)
C‐reactive protein, mg/dl	0.4 (0.4–1.1)
Serum albumin, g/dL	3.4 ± 0.5
Geriatric Nutritional Risk Index	87.7 (80.0–95.3)
Updated Charlson comorbidity index	2.0 (0.0–3.0)
Number of medications	7.0 (5.0–9.0)
Number of rehabilitation therapy, units/day	3.0 (2.0–4.0)
Barthel index score	40.0 (20.0–60.0)

Data are presented as median (interquartile range), *n* (%), or mean ± standard deviation.

*Table* 2 shows the results of the comparisons in the quadriceps echo intensity and thickness, subcutaneous fat thickness of the thigh, and BI score between hospital admission and discharge. The means of the change in quadriceps echo intensity and thickness were −2.3 ± 15.7 and 0.1 ± 0.4 cm, respectively. The medians (IQR) of the changes in subcutaneous fat thickness of the thigh and BI score were 0.0 (−0.0 to 0.1) cm and 15.0 (0.0–30.0), respectively. The quadriceps echo intensity at discharge was significantly lower than at admission. The quadriceps thickness, subcutaneous fat thickness of the thigh, and BI score at discharge were significantly higher than those at admission.

**Table 2:** Comparisons in quadriceps echo intensity and thickness, subcutaneous fat thickness of the thigh, and Barthel index score between post‐acute hospital admission and discharge

	At admission	At discharge	*P* value
Quadriceps echo intensity (grey‐scale range, 0–255)	83.8 ± 20.5	81.5 ± 20.3	0.043^a^
Quadriceps thickness, cm	1.2 ± 0.5	1.3 ± 0.6	0.004^a^
Subcutaneous fat thickness of the thigh, cm	0.4 (0.3–0.5)	0.4 (0.3–0.5)	0.007^b^
Barthel Index score	40.0 (20.0–60.0)	65.0 (40.0–85.0)	< 0.001^b^

Data are presented as mean ± standard deviation or median (interquartile range).

^a^ Paired *t* test.

^b^ Wilcoxon signed‐rank test.

*Tables* 3 and 4 show the results of multiple regression analysis for change in BI score and BI score at discharge, respectively. No multicollinearity was observed between the independent variables in both multiple regression analyses. Changes in quadriceps echo intensity and subcutaneous fat thickness of the thigh, quadriceps echo intensity at admission, sex, BI score at admission, and fracture were independently and significantly associated with the change in BI score (*R* ^2^ = 0.219, f^2^ = 0.280, statistical power = 1.000). Similarly, the same variables (i.e. changes in quadriceps echo intensity and subcutaneous fat thickness of the thigh, quadriceps echo intensity at admission, sex, BI score at admission, and fracture) were independently and significantly associated with BI score at discharge (*R* ^2^ = 0.602, f^2^ = 1.513, statistical power = 1.000). Change in quadriceps thickness was not independently and significantly associated with the change in BI score and BI score at discharge.

**Table 3:** Multiple regression analysis for change in Barthel index score

	B	SE	95% Confidence interval of B	β	VIF	*P* value
Quadriceps echo intensity at admission	−0.46	0.12	−0.69, −0.23	−0.48	3.55	<0.001
Change in quadriceps echo intensity	−0.32	0.11	−0.54, −0.09	−0.25	1.98	0.006
Quadriceps thickness at admission	−1.51	4.70	−10.77, 7.75	−0.04	2.87	0.748
Change in quadriceps thickness	4.42	3.78	−3.04, 11.87	0.09	1.43	0.244
Subcutaneous fat thickness of the thigh at admission	−14.38	7.63	−29.42, 0.66	−0.16	1.80	0.061
Change in subcutaneous fat thickness of the thigh	−28.42	11.23	−50.57, −6.28	−0.19	1.36	0.012
Age	0.13	0.19	−0.25, 0.50	0.05	1.26	0.506
Sex	−6.31	2.96	−12.15, −0.46	−0.16	1.34	0.035
Days from onset disease	0.03	0.03	−0.03, 0.09	0.08	1.31	0.268
Barthel Index score at admission	−0.27	0.07	−0.40, −0.14	−0.31	1.40	< 0.001
Disease						
Stroke	(Reference)	—	—	—	—	—
Fracture	9.25	4.13	1.12, 17.39	0.23	2.55	0.026
Pneumonia	0.04	4.97	−9.76, 9.83	0.00	1.99	0.994
Others	1.45	4.30	−7.03, 9.94	0.03	2.25	0.736

B, partial regression coefficient; SE, standard error; β, standardized partial regression coefficient; VIF, variance inflation factor.

**Table 4:** Multiple regression analysis for Barthel index score at discharge

	B	SE	95% Confidence interval of B	β	VIF	*P* value
Quadriceps echo intensity at admission	−0.46	0.12	−0.69, −0.23	−0.34	3.55	< 0.001
Change in quadriceps echo intensity	−0.32	0.11	−0.54, −0.09	−0.18	1.98	0.006
Quadriceps thickness at admission	−1.51	4.70	−10.77, 7.75	−0.03	2.87	0.748
Change in quadriceps thickness	4.42	3.78	−3.04, 11.87	0.06	1.43	0.244
Subcutaneous fat thickness of the thigh at admission	−14.38	7.63	−29.42, 0.66	−0.12	1.80	0.061
Change in subcutaneous fat thickness of the thigh	−28.42	11.23	−50.57, −6.28	−0.14	1.36	0.012
Age	0.13	0.19	−0.25, 0.50	0.03	1.26	0.506
Sex	−6.31	2.96	−12.15, −0.46	−0.11	1.34	0.035
Days from onset disease	0.03	0.03	−0.03, 0.09	0.06	1.31	0.268
Barthel index score at admission	0.73	0.07	0.60, 0.86	0.61	1.40	< 0.001
Disease						
Stroke	(Reference)	—	—	—	—	—
Fracture	9.25	4.13	1.12, 17.39	0.17	2.55	0.026
Pneumonia	0.04	4.97	−9.76, 9.83	0.00	1.99	0.994
Others	1.45	4.30	−7.03, 9.94	0.02	2.25	0.736

B, partial regression coefficient; SE, standard error; β, standardized partial regression coefficient; VIF, variance inflation factor

## Discussion

This study confirmed the decrease of intramuscular adipose tissue and increase of muscle mass of the quadriceps in older inpatients during the hospital stay. In addition, the main results of our study indicated that decrease of intramuscular adipose tissue of the quadriceps in older inpatients is related to the recovery of ADL than increase of muscle mass.

Previous studies reported that an increased intramuscular adipose tissue in the quadriceps is associated with a decreased muscle strength^5^, ^6^, ^7^ and decreased sit‐up–sit‐down^5^, ^8^ and gait abilities^6^, ^10^, ^11^ than the loss of muscle mass. Our results also indirectly support these findings. Measurement of muscle mass does not always reflect the actual muscle mass because the area of muscle mass includes both the muscle and intramuscular adipose tissue,^1^, ^14^ which potentially leads to an overestimation of the muscle mass. These factors might have influenced our results.

Considering our results, intervention for intramuscular adipose tissue of the quadriceps may be important for improving ADL in older inpatients. A recent study^28^ reported that physical activity and nutritional supplementation (whey protein and vitamin D) decreased the intramuscular adipose tissue in the thigh in community‐dwelling, mobility‐limited older people. In addition, another study^29^ confirmed the presence of reduced intramuscular adipose tissue in the lumbar muscles of patients with liver cirrhosis whose serum albumin concentration was improved following supplementation with branched‐chain amino acids. We consider that an intervention aimed at improving physical activity and nutritional status is needed to decrease the intramuscular adipose tissue of the quadriceps in older inpatients. A further randomized controlled trial will be needed.

The European Working Group of Sarcopenia in Older People 2 suggested the importance of assessing not only muscle mass but also intramuscular adipose tissue when evaluating muscle quality.^30^ In addition, the Position Statements of the Sarcopenia Definition and Outcomes Consortium suggested that muscle mass measured using dual‐energy X‐ray absorptiometry is not a good predictor of mobility limitations, falls, hip fractures, and mortality.^31^ Our results supported these proposals. We consider that assessing intramuscular adipose tissue of the quadriceps is important for predicting recovery of ADL in older inpatients.

Targeting the quadriceps is a strong point of this study. More recently, the International Society of Physical and Rehabilitation Medicine special interest group on sarcopenia included the quadriceps thickness as an indicator of muscle mass in the diagnosis criteria of sarcopenia.^32^ Furthermore, intramuscular adipose tissue of the quadriceps is related to swallowing ability,^33^, ^34^ nutritional status,^35^ aerobic capacity,^36^ and occurrence of hospital‐associated complications (i.e. delirium, functional decline, incontinence, falls, pressure injuries, and nosocomial infections).^37^ Therefore, targeting the quadriceps to determine the longitudinal relationship between intramuscular adipose tissue and ADL is considered reasonable.

This study has two limitations. First, we were not able to examine the longitudinal relationship between intramuscular adipose tissue of the quadriceps and ADL according to sex because of the small sample size of this study. However, a previous study reported that there is no sex difference in perilipin2 expression which indicator of intramuscular adipose tissue in older patients.^38^ In addition, greater intramuscular adipose tissue of the quadriceps at post‐acute hospital admission has been shown to be associated with worse recovery of ADL than loss of muscle mass in both older male and female inpatients.^15^ Taken together, no sex difference in the longitudinal relationship between intramuscular adipose tissue of the quadriceps and ADL might be observed. To reveal them, future larger studies will be needed. Second, a BI score <60 is considered a severely dependent condition of daily living.^39^ Considering the median (IQR) of the BI score at the admission of the participants was 40.0 (20.0–60.0); almost all participants in this study were in this condition. In other words, our results were obtained from participants who had severely dependent daily living. Different findings may be confirmed when groups with other characteristics are targeted.

## Conclusions

Our study indicates that decrease of intramuscular adipose tissue of the quadriceps is related to recovery of ADL than increase of muscle mass in older inpatients. Intramuscular adipose tissue of the quadriceps in older inpatients is considered to be a predictor for the recovery of ADL, and intervening for intramuscular adipose tissue may be important for improving ADL in older inpatients.

## Conflict of interest

The authors declare that there is no conflict of interest.

## Funding

This work was supported by JSPS KAKENHI (grant numbers JP17K18294 and JP20K19661).
